# Neuroendocrine carcinomas of the larynx: a review of current literature and case analysis

**DOI:** 10.1093/jscr/rjaf410

**Published:** 2025-06-24

**Authors:** Ingrid Raponi, Rossana Moi, Alessandra Perfetti, Angela Palma, Silvana Giacinti, Michele Battista, Piero Luigi Alò, Cecilia Nisticò, Andrea Marzetti

**Affiliations:** Department of Otolaryngology Head and Neck Surgery, Fabrizio Spaziani Hospital, 03100 Frosinone, Italy; Department of Otolaryngology Head and Neck Surgery, Fabrizio Spaziani Hospital, 03100 Frosinone, Italy; Department of Pathology, F. Spaziani Hospital, 03100 Frosinone, Italy; Department of Oral and Maxillo-Facial Sciences, Sapienza University of Rome, Via Caserta 6, 00161, Rome, Italy; Department of Oncology, F. Spaziani Hospital, 03100 Frosinone, Italy; Department of Radiotherapy, F. Spaziani Hospital, 03100 Frosinone, Italy; Department of Pathology, F. Spaziani Hospital, 03100 Frosinone, Italy; Department of Oncology, F. Spaziani Hospital, 03100 Frosinone, Italy; Department of Otolaryngology Head and Neck Surgery, Fabrizio Spaziani Hospital, 03100 Frosinone, Italy

**Keywords:** neuroendocrine carcinoma, laryngeal cancer, small cell carcinoma, large cell neuroendocrine carcinoma, immunohistochemistry

## Abstract

Neuroendocrine carcinomas (NECs) of the larynx are rare malignancies with aggressive behavior, histopathological diversity, and poor prognosis. Their management remains challenging due to limited case reports and the lack of standardized treatment guidelines. We conducted a comprehensive literature review to summarize classification, diagnosis, and treatment approaches for laryngeal NECs. Additionally, we present a representative case of a 64-year-old male patient diagnosed with large cell NEC of the larynx, treated with surgical resection and adjuvant radiotherapy, with long-term follow-up. Laryngeal NECs require a multidisciplinary approach, and treatment should be tailored based on tumor grade, stage, and histopathological subtype. Advances in molecular profiling and targeted therapies may improve patient outcomes in the future. Due to their rarity, further prospective studies and international collaborations are needed to establish evidence-based treatment guidelines.

## Introduction

Neuroendocrine carcinomas (NECs) of the larynx are a rare and heterogeneous group of malignant tumors characterized by neuroendocrine differentiation and aggressive clinical behavior. Representing less than 1% of all malignant tumors of the larynx, these tumors pose significant diagnostic and therapeutic challenges because of their variable histopathologic presentation and high metastatic potential [1]. The World Health Organization (WHO) classifies laryngeal NECs into three main categories: well-differentiated neuroendocrine tumors (WD-NETs), moderately differentiated neuroendocrine carcinomas (MD-NECs), and poorly differentiated neuroendocrine carcinomas (PD-NECs), the latter comprising small-cell NEC (SCNEC) and large-cell NEC (LCNECs) variants [2]. These neoplasms share histologic and immunohistochemical features with NECs of the lung and gastrointestinal tract, although their different anatomic location affects clinical management and prognosis [3].

The etiology of laryngeal NECs remains unclear, and clinically, these tumors often present nonspecific symptoms such as dysphonia, dysphagia, and airway obstruction, resulting in diagnostic delays. Early diagnosis and accurate classification are critical to optimize patient outcomes. Immunohistochemical analysis plays a key role in distinguishing these malignant tumors from other laryngeal cancers. Current treatment strategies for laryngeal NECs vary according to histologic subtype and stage of disease [4]. Surgical resection remains the cornerstone for localized WD-NETs and MD-NECs, whereas high-grade PD-NECs often require multimodal approaches, including chemotherapy and radiotherapy [3]. This review aims to summarize the latest advances in the classification, diagnosis, and treatment of laryngeal NECs, integrating knowledge from recent literature and presenting an analysis of representative cases.

## Case report

**Figure 1:**
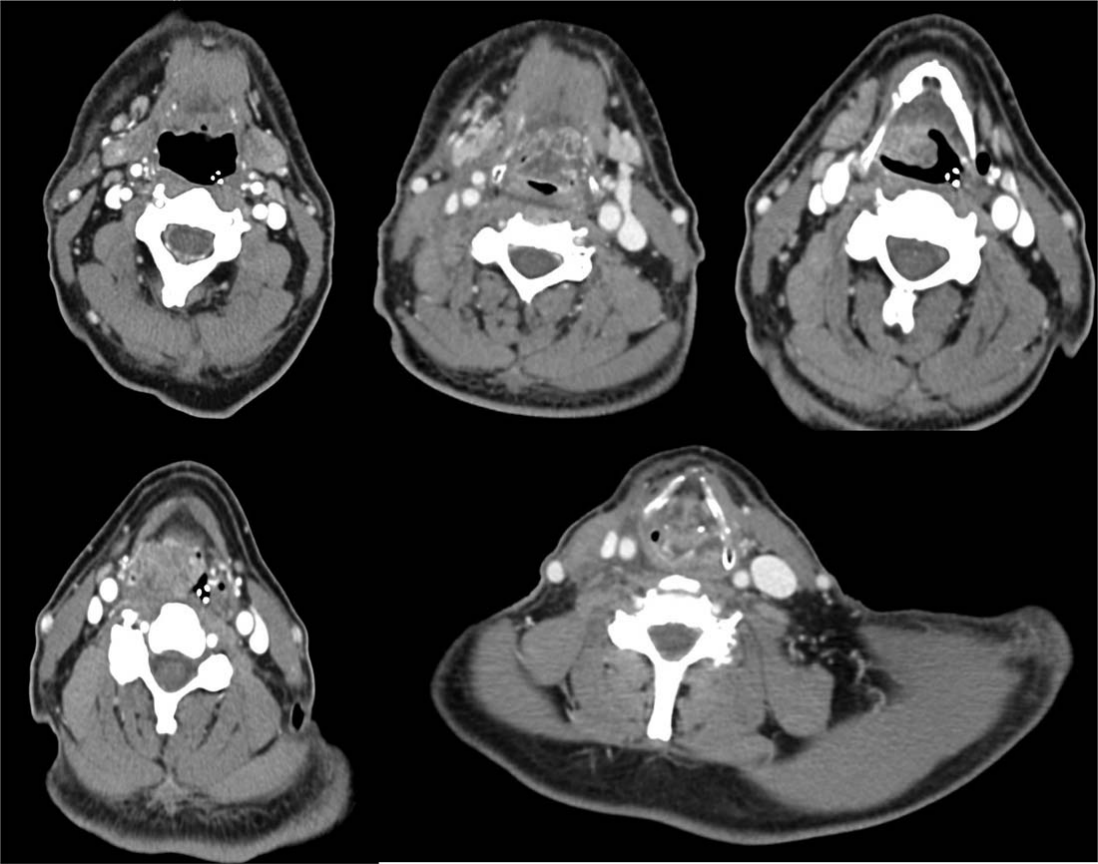
A contrast-enhanced CT scan of the head and neck, in coronal view, revealed a heterogeneous mass in the right supraglottic region, involving the aryepiglottic fold and piriform sinus with full-thickness infiltration. The lesion extended to the paralaryngeal and parapharyngeal spaces, causing obliteration of adipose planes and thyroid cartilage invasion and severe airway reduction.

**Figure 2:**
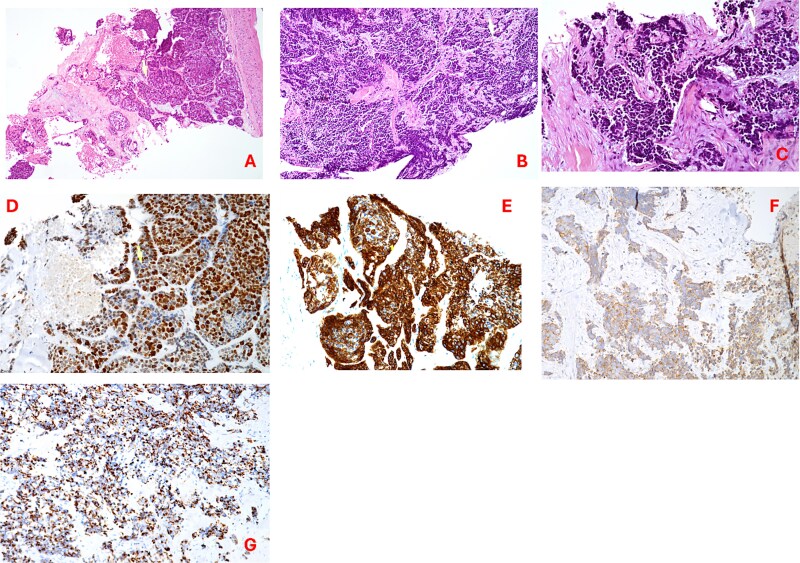
(A–C) Hematoxylin–eosin staining (20×) showing irregular trabeculae and nests of neoplastic cells interspersed with fibrous connective tissue; tumor cells exhibit nuclear pleomorphism, hyperchromasia, a high nuclear-to-cytoplasmic ratio, areas of necrosis and hemorrhage; (D) Ki67 proliferation index; (E) synaptophysin immunostaining (20×); (F) chromogranin a immunostaining (20×); (G) cytokeratin AE1/AE3 (CKAE1/AE3) staining (20×).

A 64-year-old male, a former alcohol user and non-smoker, presented with a two-month history of painful dysphagia and mild dyspnea. Fiberoptic laryngoscopy revealed an exophytic mass arising from the right aryepiglottic fold within the supraglottic region. The true vocal cords were morphologically and functionally normal, though the airway was narrowed due to mass effect. Contrast-enhanced computed tomography (CT) of the head, neck, and chest revealed a heterogeneous lesion involving the right aryepiglottic fold and piriform sinus, with full-thickness infiltration and extension to paralaryngeal and parapharyngeal spaces. Adipose planes were obliterated and there was evidence of thyroid cartilage invasion (Fig. 1). Bilateral submandibular lymph nodes appeared slightly enlarged but structurally preserved. No distant metastases were detected on thoracic CT or liver ultrasound. The patient underwent urgent tracheotomy followed by biopsy, initially suggestive of poorly differentiated squamous cell carcinoma. After multidisciplinary Tumor Board evaluation, the patient was scheduled for right pharyngectomy, partial resection of the thyroid cartilage, resection of the lesion en bloc, and multilayered pharyngeal wall reconstruction. Ipsilateral neck dissection (levels II–IV) was also performed. Histopathological examination confirmed a PD LCNEC, with ulceration, necrosis, and extensive infiltration. Microscopy showed nests and trabeculae of atypical cells with a high nuclear-to-cytoplasmic ratio, nuclear pleomorphism, and areas of necrosis and hemorrhage (Fig. 2A–C). Immunohistochemistry demonstrated: high proliferative index (~80%) for Ki67 (Fig. 2D), diffuse strong positivity for Synaptophysin (Fig. 2E), cytoplasmic positivity for Chromogranin A (Fig. 2F), paranuclear dot-like positivity for CK AE1/AE3 (Fig. 2G). One lymph node at level IV showed metastatic involvement. Final staging was pT1 pN0 pMx, stage I, G3 (UICC 2017). Based on Tumor Board recommendations, the patient received adjuvant radiotherapy with a total dose of 63 Gy. At 5-year follow-up, the patient remains disease-free with no clinical or radiological evidence of recurrence. The rarity of LCNEC of the larynx, combined with the achievement of a complete surgical resection followed by adjuvant radiotherapy and a 5-year disease-free survival, makes this case particularly valuable in highlighting the potential for long-term control in selected patients.

## Discussion

Neuroendocrine carcinomas of the larynx (LNECs) are exceedingly rare malignancies, with only a few hundred cases reported in the literature, predominantly as isolated case reports or small series [5]. These tumors exhibit a wide spectrum of histopathological differentiation, which significantly influences prognosis and treatment strategy. The latest systematic review by Strojan *et al*. (2019) [6] analyzed 273 cases reported in the literature and provided a comprehensive comparison between SCNEC and LCNEC. WD-NETs are indolent, low-proliferation lesions expressing neuroendocrine markers such as chromogranin A, synaptophysin, and CD56, and although generally slow-growing, they carry a risk of regional or distant spread. MD-NECs are more aggressive, with increased mitotic activity and focal necrosis, and typically require surgical resection, often combined with adjuvant radiotherapy [1]. PD-NECs, including SCNEC and LCNEC variants, represent the most aggressive forms, with dismal 5-year disease-specific survival rates below 20%. While both SCNEC and LCNEC are classified as PD-NECs with high mitotic indices, they differ significantly in clinical behavior and treatment responsiveness. SCNEC is more frequent and typically associated with systemic relapses and early distant spread, often warranting systemic chemotherapy even at early stages, based on regimens used for small cell lung cancer. LCNEC, on the other hand, affects older patients and more often involves the supraglottic larynx; it may present with a slightly more indolent course and shows lower chemosensitivity, making surgical resection a more viable initial strategy when feasible. Despite these differences, both subtypes retain high metastatic potential and poor long-term survival rates [6]. Management lacks standardized protocols and is largely guided by treatment principles derived from pulmonary NECs [7]. Surgical excision remains the mainstay for low and intermediate-grade tumors, whereas high-grade NECs demand a multimodal approach including platinum-based chemotherapy, radiotherapy, and, selectively, surgery. Immunotherapy, particularly checkpoint inhibitors, is being explored for high-grade disease, especially in cases with molecular alterations such as TP53 and RB1 mutations or MYC amplification [8]. Systemic recurrence is the predominant failure pattern in LNECs, reinforcing the need for early systemic intervention when feasible. Prognosis is primarily determined by tumor grade, mitotic index, nodal status, and histological subtype. Table 1 summarizes treatment approaches and estimated 5-year survival rates for laryngeal NECs based on tumor grade and histologic subtype (WD, MD, and PD-NECs, including SCNEC and LCNEC).

**Table 1:** Staging, treatment, and survival rate table according to 8th edition of the AJCC TNM staging system (2017) for laryngeal cancer

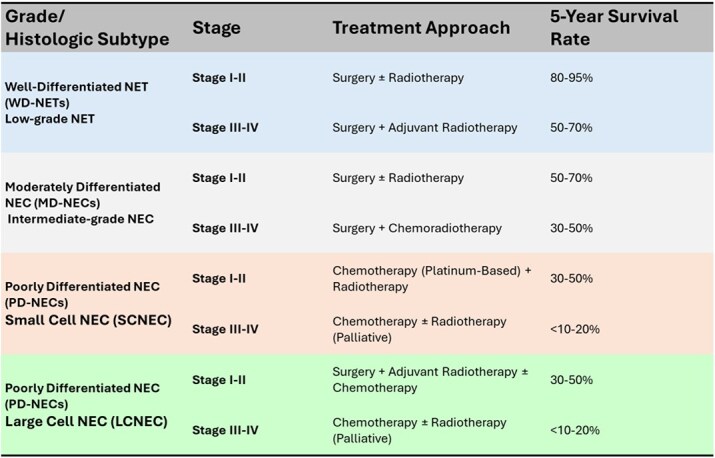

Recent developments in the management of extrapulmonary NECs, including those of the head and neck, have focused on the potential role of immune checkpoint inhibitors. A 2021 review by Stelwagen *et al*. underscored the limited but growing evidence for the use of immunotherapy in high-grade NECs, particularly in patients exhibiting PD-L1 expression or microsatellite instability [7]. Additionally, a 2023 review on gastrointestinal NENs by Pan *et al.* reported promising outcomes with combination immune checkpoint blockade, including objective response rates of up to 26% in high-grade disease [9]. Although these data derive from non-head and neck sites, they provide a biological rationale for exploring similar approaches in rare aggressive NECs of the larynx. The DART trial (SWOG S1609; ClinicalTrials.gov Identifier: NCT02834013) includes a cohort for extrapulmonary NECs, though no results specific to these tumors have yet been reported. These findings collectively highlight the need for prospective studies and molecular profiling to guide future immune-based strategies.

Due to their rarity and the heterogeneity of clinical behavior, international collaboration and prospective studies are necessary to define optimal treatment strategies and improve outcomes. Laryngeal NECs represent a challenging and under-recognized entity with distinct histopathological subtypes and variable clinical behavior. While WD-NETs and MD-NECs can often be managed surgically with favorable outcomes, PD-NECs require an aggressive multimodal approach due to their high metastatic potential. Advances in molecular diagnostics and immunotherapy hold promise for improving outcomes in patients with high-grade disease. Future research should focus on standardizing diagnostic criteria, optimizing treatment protocols, and exploring novel targeted therapies to improve patient outcomes.
